# Supplementary figures and images for: VOE: automated analysis of variant epitopes of SARS-CoV-2 for the development of diagnostic tests or vaccines for COVID-19
Source: PeerJ. 2024 Jun 19;12:e17504. doi: 10.7717/peerj.17504 (PMC11193398; doi:10.7717/peerj.17504)

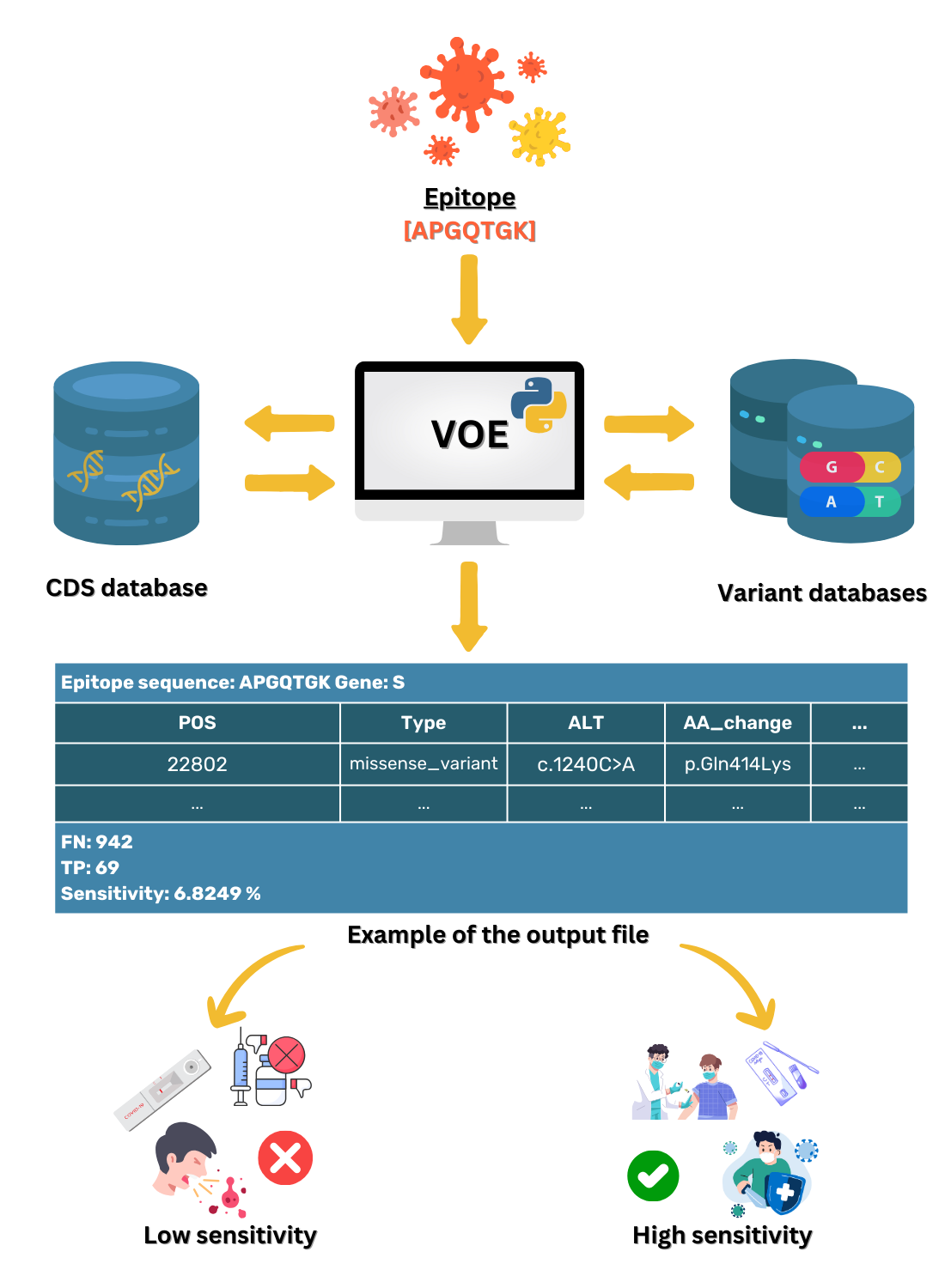

Supplement: Supplemental Information 2 — A user simply enters an epitope, then VOE will report all variants of the epitope and the sensitivity for the epitope. [file peerj-12-17504-s002.png]
